# TE-SCALE: a comprehensive database for exploring transposable element expression across human cancers at single-cell resolution

**DOI:** 10.1093/nar/gkaf1235

**Published:** 2025-11-26

**Authors:** Xini Meng, Zhi Nie, Qifei Wang, Yiwen Hu, Yulan Deng, Na Ai, Zheng Huang, Yun Li, Yang Yuan, Jingfa Xiao, Jingyao Zeng, Guochao Li, Lan Jiang

**Affiliations:** China National Center for Bioinformation, Beijing 100101, China; Beijing Institute of Genomics, Chinese Academy of Sciences, Beijing 100101, China; University of Chinese Academy of Sciences, Beijing 100049, China; China National Center for Bioinformation, Beijing 100101, China; Beijing Institute of Genomics, Chinese Academy of Sciences, Beijing 100101, China; University of Chinese Academy of Sciences, Beijing 100049, China; National Genomics Data Center, China National Center for Bioinformation, Beijing 100101, China; China National Center for Bioinformation, Beijing 100101, China; Beijing Institute of Genomics, Chinese Academy of Sciences, Beijing 100101, China; University of Chinese Academy of Sciences, Beijing 100049, China; China National Center for Bioinformation, Beijing 100101, China; Beijing Institute of Genomics, Chinese Academy of Sciences, Beijing 100101, China; University of Chinese Academy of Sciences, Beijing 100049, China; Department of Thoracic Surgery and Institute of Thoracic Oncology, West China Hospital of Sichuan University, Chengdu 610041, China; Western China Collaborative Innovation Center for Early Diagnosis and Multidisciplinary Therapy of Lung Cancer, Sichuan University, Chengdu 610041, China; China National Center for Bioinformation, Beijing 100101, China; Beijing Institute of Genomics, Chinese Academy of Sciences, Beijing 100101, China; Beijing Life Science Academy, Lutuan East Road, Beijing 102200, China; Key Laboratory of Tobacco Biological Effects, Lutuan East Road, Beijing 102200, China; China National Center for Bioinformation, Beijing 100101, China; Beijing Institute of Genomics, Chinese Academy of Sciences, Beijing 100101, China; University of Chinese Academy of Sciences, Beijing 100049, China; China National Center for Bioinformation, Beijing 100101, China; Beijing Institute of Genomics, Chinese Academy of Sciences, Beijing 100101, China; University of Chinese Academy of Sciences, Beijing 100049, China; University of Chinese Academy of Sciences, Beijing 100049, China; Institute for Stem Cell and Regeneration, Chinese Academy of Sciences, Beijing 100101, China; China National Center for Bioinformation, Beijing 100101, China; Beijing Institute of Genomics, Chinese Academy of Sciences, Beijing 100101, China; University of Chinese Academy of Sciences, Beijing 100049, China; National Genomics Data Center, China National Center for Bioinformation, Beijing 100101, China; China National Center for Bioinformation, Beijing 100101, China; Beijing Institute of Genomics, Chinese Academy of Sciences, Beijing 100101, China; National Genomics Data Center, China National Center for Bioinformation, Beijing 100101, China; China National Center for Bioinformation, Beijing 100101, China; Beijing Institute of Genomics, Chinese Academy of Sciences, Beijing 100101, China; University of Chinese Academy of Sciences, Beijing 100049, China; China National Center for Bioinformation, Beijing 100101, China; Beijing Institute of Genomics, Chinese Academy of Sciences, Beijing 100101, China; University of Chinese Academy of Sciences, Beijing 100049, China; College of Future Technology College, University of Chinese Academy of Sciences, Beijing 100049, China

## Abstract

Transposable elements (TEs) are repetitive DNA sequences typically silenced in normal tissues. Their dysregulation in cancer can significantly impact oncogene activation and tumorigenesis. However, due to their repetitive nature, TEs are often excluded from gene-centric single-cell analyses. Although recent advances have enabled single-cell TE quantification, a systematic resource for exploring single-cell TE dynamics in cancer has remained lacking. To address this, we developed TE-SCALE (https://ngdc.cncb.ac.cn/te-scale/), a single-cell database for integrative analysis and visualization of TE expression across human cancers. It incorporates over 1.3 million cells from 330 samples across 20 cancer types and 12 tissue origins. Built on our in-house pipeline scTEfinder, TE-SCALE provides a comprehensive pan-cancer TE expression atlas, enabling multi-scale exploration from tissue to cell population, supported by well-curated TE annotations. The platform offers three key analytical modules: differential TE expression, TE-gene co-expression network, and functional enrichment analysis. A user-friendly web interface supports flexible browsing, searching, analysis, and data download. Notably, TE-SCALE identifies tumor-specific TEs preferentially expressed in particular cancer types or disease states, underscoring their potential as biomarkers for diagnosis, monitoring, and immunotherapeutic targeting. Collectively, TE-SCALE provides an essential resource for decoding TE biology in cancer and expedites its translation into clinical applications.

## Graphical Abstract

**Graphical Abstract:**
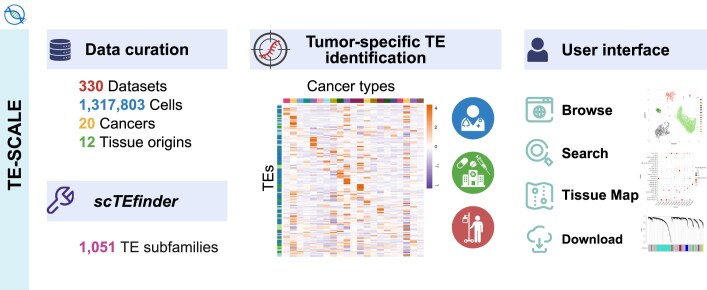

## Introduction

Transposable elements (TEs) are repetitive DNA sequences that comprise approximately 45% of the human genome [1, 2]. Based on their transposition mechanisms, TEs are classified into two major classes: retrotransposons and DNA transposons [3]. Retrotransposons represent the predominant class of TEs in humans and propagate via an RNA intermediate through a “copy-and-paste” mechanism mediated by reverse transcription. This class encompasses four principal subclasses: long interspersed nuclear elements (LINEs), short interspersed nuclear elements (SINEs) such as Alu, SINE-variable number tandem repeat-Alu (SVA) elements, and long terminal repeat (LTR) elements. In contrast, DNA transposons mobilize via a “cut-and-paste” mechanism, in which the element is excised from its donor site and reinserted into a new genomic locus as a DNA fragment, typically catalyzed by transposases.

Although the majority of TEs have lost autonomous transposition capacity due to accumulated mutations, their genomic remnants retain significant regulatory potential, influencing epigenetic and transcriptional networks [4–6]. In normal tissues, this regulatory activity is stringently suppressed to maintain genomic stability. However, in cancer, widespread epigenetic dysregulation often leads to the loss of TE repression, resulting in their aberrant transcriptional reactivation and even tumorigenesis [7–10]. Reactivated TEs might disrupt coding sequences or regulatory regions through insertional mutagenesis [5], [9]. Furthermore, they could be co-opted by the host genome as cryptic promoters or enhancers, driving oncogene overexpression [11]. Notably, widespread activation of TE-derived promoters can generate chimeric transcripts that fuse TE sequences with downstream genes, potentially encoding TE-gene fusion proteins or truncated TE-encoded polypeptides [12–14]. These TE-derived products constitute an important source of neoantigens and are immunologically active in various cancer types. Genomic reanalysis reveals that 30.4% of major histocompatibility complex (MHC) class I-associated tumor-specific antigens originate from TEs [14, 15]. In lung adenocarcinomas, aberrant expression of HERV-K102 induces antibody responses and correlates with transcriptional signatures of CD8^+^ T cells and natural killer cells, highlighting its potential as an immunotherapeutic target [16].

TEs have also been shown to possess diagnostic and prognostic value in cancers [17, 18]. For instance, HERV-E expression is restricted to tumors in clear cell renal cell carcinoma, which can be detected even at the earliest stages of disease [19]. LINE-1 open reading frame 1 and 2 proteins (ORF1p and ORF2p) have been used as cancer biomarkers for non-invasive screening and clinical prediction in breast cancer [20, 21], colorectal cancer [21, 22], hepatocellular carcinoma [23], and ovarian cancer [22, 24]. These characteristics underscore the potential of TEs as promising biomarkers for cancer diagnosis and monitoring, as well as targets for cancer immunotherapy [18, 25, 26].

Utilizing large-scale genomic resources such as The Cancer Genome Atlas (TCGA) and the Genotype-Tissue Expression (GTEx) project, several studies have developed tools and conducted systematic pan-cancer analyses of TE expression [10, 12–14]. More recently, advanced bioinformatics methods have been introduced to quantify TE expression in single-cell RNA sequencing (scRNA-seq) data. Among them, scTE is the first and most widely adopted pipeline for single-cell TE analysis, which estimates TE subfamily expression by assigning ambiguous reads to consensus “metagenes” representing TE subfamilies [27]. Subsequent tools such as SoloTE [28] and MATES [29] enable locus-level quantification of TE expression. This improved resolution facilitates the characterization of TE heterogeneity in the tumor microenvironment (TME) [30–32]. However, the existing TE expression databases in cancer, such as CancerHERVdb [33] and ERVcancer [34], are restricted to the specific TE class and lack single-cell resolution. scARE, the only currently available single-cell TE resource to our knowledge, focuses on neurodegenerative diseases and encompasses a relatively limited range of datasets [35]. Consequently, a comprehensive database for exploring TE expression profiles across diverse cancer types at single-cell resolution remains an unmet need.

To address this, we present TE-SCALE (Transposable Element Single-Cell Analysis for pan-cancer Landscape Exploration), a comprehensive and user-friendly database designed for the systematic analysis and visualization of TE expression patterns across human cancers at single-cell resolution. It is constructed from multiple publicly available scRNA-seq datasets, comprising 1 317 803 high-quality cells from 330 samples across 20 cancer types and 12 tissue origins. Leveraging an in-house streamlined pipeline, scTEfinder, TE-SCALE enables robust quantification of 1051 curated TE subfamilies, integrates gene and TE expression profiles, and provides accurate cell-type annotation. The user-friendly web interface allows researchers to interactively explore TE transcriptomics across diverse malignancies. It integrates downstream analytical modules with visualized results, featuring differential TE expression, TE-gene co-expression networks, and functional enrichment analysis. As an actively maintained and regularly updated resource, TE-SCALE aims to serve as a powerful tool to elucidate the landscape of dysregulated TE expression and potential regulatory mechanisms in tumor cells and other components of the TME, thereby providing a valuable foundation for the development of TE-targeted clinical applications.

## Material and methods

### Data collection and curation

We curated a total of 330 scRNA-seq datasets from tumor and normal samples, all generated using the 10x Genomics platform [36]. Raw sequencing reads were retrieved from publicly available repositories, including CancerSCEM (version 1.0) [37, 38], GEO [39], ArrayExpress [40], GSA [41, 42], GSA-Human [41], [42], and other public resources, including data directly provided by original authors [43]. (Supplementary Table S1). These samples cover 12 tissue origins and 20 cancer types, with the database encompassing nearly all matched normal tissues corresponding to the selected cancer types. Tumor samples comprise 73.9% of the dataset, while normal samples account for the remaining 26.1%. The metadata, such as donor identity, age, sampling site, and clinical stage, were also collected when available.

TE metadata were retrieved from the Dfam database [44] to support browsing and classification, as well as statistical analysis of genome annotations. The age of individual TEs was estimated by dividing the number of substitutions from the consensus sequence by the human mutation rate of 2.2 × 10^-9^ substitutions per base pair per year [45, 46]. Additionally, we manually curated a collection of published studies that explored TEs as potential targets for cancer immunotherapy using various strategies (Supplementary Table S2). These curated resources complement the computational analyses in the database and provide users with a valuable reference for evaluating the clinical relevance of candidate TEs.

### Gene and TE expression quantification

TE repetitiveness poses challenges for single-cell quantification [47]. To address this, we developed scTEfinder—an accurate and automated pipeline for TE quantification at the subfamily level from raw scRNA-seq data—using scTE as the core TE quantification module. This pipeline has been systematically applied to all datasets in TE-SCALE, ensuring standardized, reproducible, and high-quality TE expression profiling across diverse samples and sequencing platforms.

*Read Mapping*. Raw sequencing reads were aligned to the human reference genome (hg38) using STARsolo, a single-cell module built into the STAR aligner (version 2.7.11b) [48, 49]. To enhance the sensitivity and accuracy of TE detection, mapping parameters were carefully optimized to retain multi-mapping reads, using the following settings: –outFilterMultimapNmax 100, –winAnchorMultimapNmax 100, –outMultimapperOrder Random, –runRNGseed 777, and –outSAMmultNmax 1.

*Quality Control*. Low-quality cells were filtered out based on the following criteria: gene count > 500, unique molecular identifier (UMI) count > 1000, and mitochondrial gene read proportion < 10%. Putative doublets were identified and removed using DoubletFinder (version 2.0.4) [50]. High-confidence cell barcodes passing quality control were extracted, and original BAM files were subsetted using bcsubset (v0.0.1) (https://github.com/kehrlab/bcsubset) to retain only reads from qualified cells, ensuring downstream analyses on high-quality single-cell transcriptomic data.

*Expression Quantification*. TE expression at the subfamily level was quantified using scTE (version 1.0) [27], leveraging human genome indices (hg38) that integrate TE and gene annotations. This process generated single-cell expression matrices for both genes and TE subfamilies.

In addition, we performed automated cell-type annotation using CellTypist (version 1.6.3) [51], which relies on a pretrained human cross-tissue reference model from the Human Protein Atlas (HPA) [52, 53], a resource encompassing 32 human tissues, more than 11 million cells, and 82 cell types validated by immunohistochemistry, to assign fine-grained cell-type labels for downstream analyses.

### Analysis of scRNA-seq data

We applied the Seurat package (version 4.4.0) [54] in R to process single-cell transcriptomic data and profile TE expression and cellular heterogeneity. The matrices were first normalized using the “NormalizeData” function, followed by scaling with the “ScaleData” function. The top 3000 highly variable genes and TEs were selected for dimensionality reduction via principal component analysis (PCA), and the first 30 PCs were used for cell clustering with the Louvain algorithm. Cell distributions were visualized in two-dimensional space using the uniform manifold approximation and projection (UMAP) method.

Copy number variation (CNV) inference was performed using SCEVAN (version 1.0.3) [55] to distinguish tumor cells from the normal ones. According to pre-annotated cell types and CNV-inferred tumor cells, differentially expressed genes and TEs among various cell populations were identified using the “FindAllMarkers” function in Seurat, with significance defined as adjusted *P* value < 0.05. Functional enrichment analysis was conducted via Gene Set Variation Analysis (GSVA) (version 1.52.3) [56], using MSigDB hallmark gene sets for well-defined biological processes enrichment [57, 58], and UCSC RepeatMasker annotations for TE family enrichment [59], which is based on RepeatMasker Open-3.0 (https://www.repeatmasker.org/). Tumor-specific pathways were further analyzed and identified using limma (version 3.60.4) [60].

### Integrated analysis of TE expression by tissue origin

For cancer types derived from the same tissue origin, we merged their Seurat objects and performed integrated analysis to examine shared and cancer-specific TE expression patterns. Batch effects from donors and sequencing platforms were corrected using Harmony (version 1.2.1) [61] on the PCA embeddings, which were then used for clustering and UMAP visualization following the standard analysis workflow described above.

*Differential expression analysis*. Differential expression analysis using the “FindMarkers” function in Seurat identified tumor-specific TE subfamilies, which were defined as positive markers distinguishing tumor cells specific to each cancer type from their normal counterparts within the same tissue. To reduce false discoveries, we applied two complementary strategies: a single-cell Wilcoxon rank-sum test, and a pseudobulk approach based on DESeq2 (version 1.44.0) [62, 63], with feature expression aggregated to the sample level using the “AggregateExpression” function prior to analysis.

For datasets with available clinical information, differential TE expression analysis across tumor stages was performed using the “FindMarkers” function. Tumor cells were grouped by stage within each cancer type, and the Wilcoxon rank-sum test was applied to identify stage-specific TE subfamilies.

*Co-expression network analysis*. We performed tumor-specific weighted gene co-expression network analysis (WGCNA), where “gene” broadly includes protein-coding genes and TEs, using the hdWGCNA package (version 0.3.3) [64, 65]. First, “metacells” were constructed with the “MetacellsByGroups” function in hdWGCNA, aggregating the expression profiles of the 50 nearest transcriptomically similar neighboring cells within each cell type and biological sample [66]. Next, the “SetDatExpr” function was applied to set the expression data on the metacell expression matrix of tumor cells, followed by testing various soft-thresholding powers using “TestSoftPowers” to determine the optimal soft power. The weighted co-expression network was then constructed using the “ConstructNetwork” function, resulting in the identification of distinct co-expression modules. To access module expression, we computed module eigengenes (MEs), the first PCs summarizing module-wide expression, using the “ModuleEigengenes” function. Module connectivity, representing the correlation between each gene/TE and its corresponding ME, was calculated through the “ModuleConnectivity” function.

For each module, regulatory genes and TEs were annotated with cancer-related information. Cancer-related genes included oncogenes, tumor suppressor genes (TSGs), and cancer drivers curated from OncoKB [67, 68] and Network of Cancer Genes (NCG) [69]. Tumor-specific TEs were defined based on the differential TE expression analysis described above.

*Functional enrichment analysis*. Genes and TEs for each module were extracted using the “GetModules” function in hdWGCNA. Functional enrichment analysis was then performed separately for genes and TEs using the “enricher” function from the clusterProfiler package (version 4.12.6) [70, 71], with hallmark gene sets and TE family annotations as respective references. Only terms with an adjusted *P* value < 0.05 were retained to characterize the potential function features of each module and its composition of TEs.

### Implementation of the database

The TE-SCALE database was implemented following standard web development practices. The system architecture was designed with a PostgreSQL relational database to store and manage dataset metadata, ensuring structured and scalable data handling. The frontend interface and backend server were developed using the SvelteKit framework, a modern and efficient web framework based on Svelte, enabling high-performance user interactions and dynamic content rendering. To streamline database interactions, Drizzle ORM was employed to map SvelteKit application logic to PostgreSQL operations in a type-safe manner. For efficient client-side data management, TanStack Query was integrated to handle data fetching, caching, synchronization, and real-time updates between the frontend and backend. The user interface was designed using Flowbite, a UI component library built on Tailwind CSS, ensuring a consistent and responsive visual style across devices and platforms. Interactive visualizations were implemented using ECharts and plotly.js, enabling dynamic and customizable data plots such as UMAP embeddings and violin plots. Tabular data representations were rendered using TanStack Table, a flexible and high-performance data table library that supports sorting, filtering, and pagination. All backend computational tasks were executed via custom R scripts.

## Database contents and usage

### Overview of TE-SCALE database

**Figure 1.**
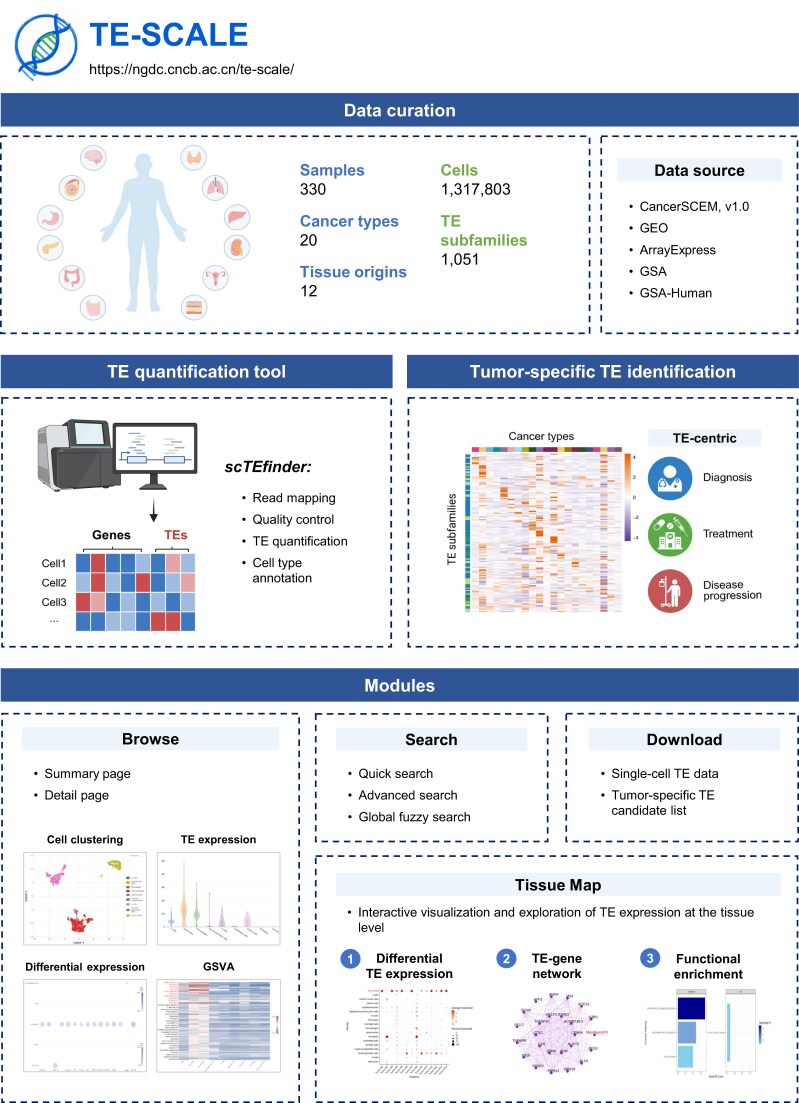
A schematic diagram illustrating the key contents and functional modules of TE-SCALE. The database integrates curated scRNA-seq data from multiple cancer types, employs the scTEfinder pipeline for TE quantification, and supports tumor-specific TE identification with clinical relevance. It offers four modules for user interaction and data retrieval: browsing, search, an interactive “Tissue Map” for multiscale TE exploration, and download of single-cell TE data. Some schematic elements were created with BioRender.com.

TE-SCALE was designed to systematically uncover the transcriptional dynamics and biological relevance of TEs across human cancers at single-cell resolution (Fig. 1), addressing their long-standing underrepresentation in traditional gene-centric analyses. A total of 330 scRNA-seq samples from 20 cancer types and 12 tissue origins were curated and reanalyzed, with essential metadata (e.g. biosample characteristics, sequencing platforms, clinical information) systematically compiled. After rigorous quality control, 1 317 803 high-quality cells were retained. Major cancers such as lung cancer, pancreatic cancer, and thyroid cancer are well represented, and samples and cells from multiple other cancer types are also included in the database (Supplementary Fig. S1). To ensure consistent and scalable quantification of TEs, a customized pipeline, scTEfinder, was applied across all datasets. The expression profiles of 1051 well-annotated TE subfamilies were then obtained, spanning five major TE classes — LTRs, LINEs, SINEs, SVAs, and DNA transposons — and mapped across 81 fine-grained cell types. To our knowledge, TE-SCALE offers the broadest coverage of TE subfamilies, the highest resolution with single-cell data, and the richest dataset for cancer-related TE expression.

TE-SCALE provides a user-friendly web interface for querying, browsing, and downloading detailed information and analytical results for all samples and TE subfamilies. In particular, it features an interactive “Tissue Map” interface to facilitate tissue-level visualization and exploration of TE expression patterns. Functional modules further support differential TE expression analysis, TE-gene co-expression network construction, and pathway enrichment analysis, specifically in tumor cells. By integrative pan-cancer analysis, tumor-specific TEs were identified across different cancer types. Additionally, TE-SCALE includes a curated set of clinical studies on TEs, offering a useful resource for advancing TE’s diagnostic and immunotherapeutic potential.

### Scalable tool for single-cell TE analysis

**Figure 2.**
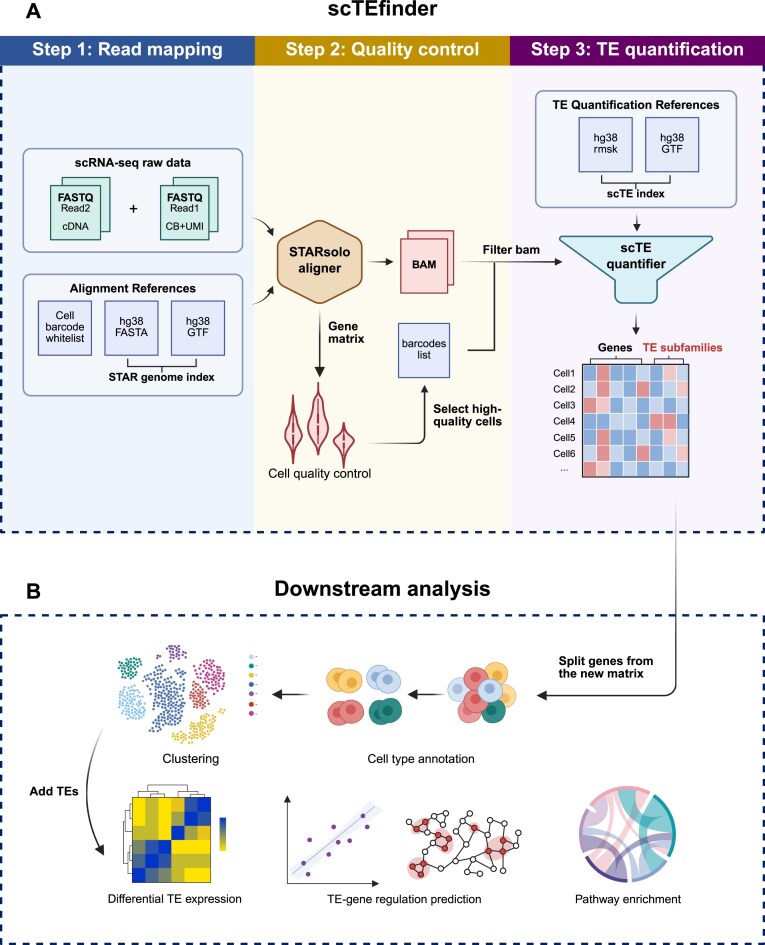
scTEfinder pipeline for single-cell TE analysis. (**A**) Workflow comprising three main steps: read mapping, quality control, and TE quantification. (**B**) Example applications of scTEfinder outputs in downstream analyses of gene and TE expression. Panels A and B were created with BioRender.com.

TE-SCALE incorporates scTEfinder, a streamlined and scalable pipeline for subfamily-level quantification of TEs at single-cell resolution with scTE software as the core TE quantification module, which is also available as a standalone tool for offline use. scTEfinder generates high-quality single-cell gene and TE expression matrices, along with cell-type annotations, directly from raw sequencing data. By default, it employs a built-in complete human reference genome with integrated gene and TE annotations. The pipeline automates three key steps: (i) Read mapping, optimized to retain multi-mapping reads that may derive from TEs; (ii) Quality control, which filters out low-quality and other aberrant reads or cells; and (iii) TE quantification, performed at the subfamily level using scTE [27] (Fig. 2A). An optional cell type annotation module leverages a pretrained multi-tissue human model to assign cell types automatically. While optimized for 10x Genomics scRNA-seq data (3′ v1/v2/v3 and 5′ chemistries), the pipeline can accommodate other single-cell platforms with suitable adjustments. Researchers can apply scTEfinder to their own datasets for accurate TE quantification and comprehensive downstream analyses compatible with Seurat [54] or Scanpy [72], including dimensionality reduction and clustering, differential expression analysis, and functional enrichment analysis (Fig. 2B). By adding TE expression into standard single-cell analysis workflows, scTEfinder expands the transcriptomic landscape, enables unified pan-cancer TE quantification, and facilitates broader applications without adding analytical complexity.

### User-friendly interfaces for data browsing and search

TE-SCALE offers an intuitive “Browse” page featuring two main interactive tables on sample data and TE subfamilies, which are organized for convenient exploration of data of interest.

**Figure 3.**
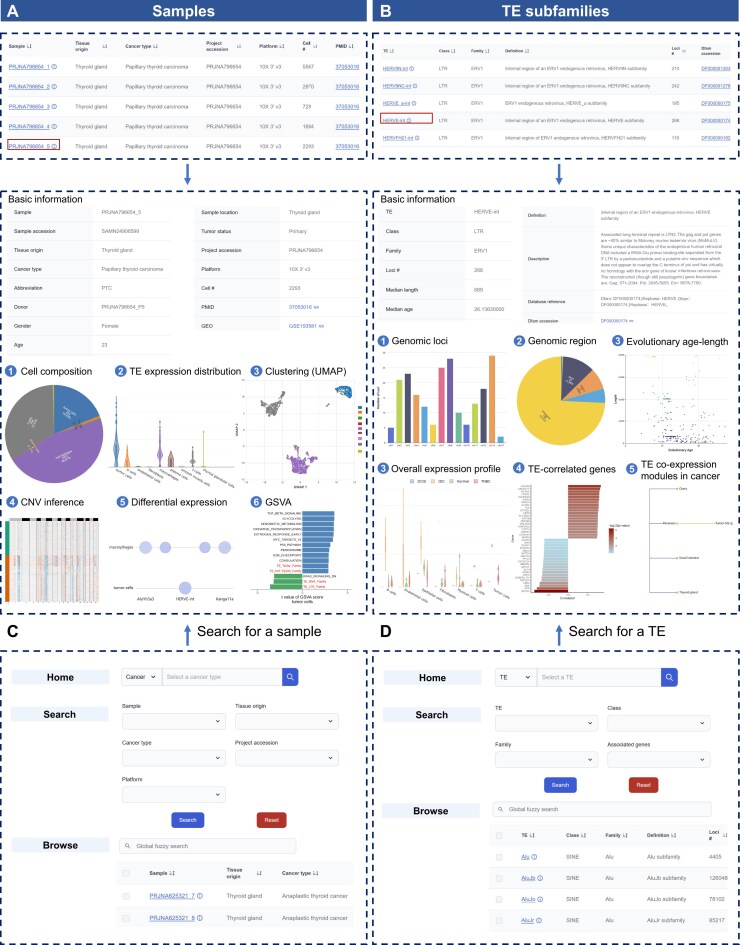
Demonstration of browsing and search interfaces in TE-SCALE. (**A**) Screenshot of the sample browsing table, showing basic information (top) and detailed sample data for PRJNA796654_5 as an example. Details include sample metadata, cell composition, TE expression distribution, clustering UMAP, CNV inference, differential expression, and GSVA-based functional enrichment (bottom). (**B**) Screenshot of the browse table of TE subfamilies with basic information and summary statistics (top) and detailed information for HERVE-int as an example, including curated annotation, intrinsic features, genomic distribution, overall expression profiles across datasets, predicted TE-gene correlations, and co-expression modules in cancer (bottom). (**C** and **D**) Interfaces for searching a specific sample (**C**) or TE (**D**) via three search channels, implemented in the “Home,” “Search,” and “Browse” pages, respectively.

A series of standardized single-cell TE analyses was conducted for each sample across multiple dimensions. The sample browsing table presents key metadata such as sample ID, tissue origin, cancer type, project accession number, sequencing platform, filtered cell count, and publication source, serving as an entry point to explore the associated data and analytical results (Fig. 3A, top). Moreover, users can access dedicated pages for each sample to obtain comprehensive metadata, extended links to public databases and publications, and a suite of analytical outputs with interactive visualizations. Included are cell type composition, TE expression distribution, UMAP-based clustering, CNV inference, differential expression, and functional enrichment analysis. A representative example page for PRJNA796654_5 [73] is shown in Fig. 3A (bottom).

TE subfamily represents another core entity in TE-SCALE. Basic information, including the subfamily name, class, family, definition, total number of genomic loci, and Dfam accession number, is listed in the TE browsing table (Fig. 3B, top). For in-depth exploration, each TE subfamily has a detailed page with curated annotations, summary statistics on genomic distribution, evolutionary age, and length, as well as overall expression profiles across single-cell datasets. In addition, TE-SCALE provides analyses of TE-correlated genes and TE co-expression modules in cancer. These resources capture co-expression networks and their associated genes across different cancer types, thereby providing a foundation for investigating TE-gene regulatory relationships and their functional roles in tumorigenesis. An example page for HERVE-int is shown in Fig. 3B (bottom).

TE-SCALE also provides multiple search methods to support efficient and user-friendly data retrieval: (i) A quick search box on the homepage enables simple queries using a specified TE subfamily name, cancer type, or tissue origin; (ii) An advanced search interface on the “Search” page allows users to perform combined queries within sample- (e.g. sample ID, tissue origin, and cancer type) or TE-related information (e.g. name, class, family, and correlated genes), allowing selection and access to specific entries; (iii) A global fuzzy search box embedded on the “Browse” page enables keyword searches within the interactive browsing tables (Fig. 3C and D). Meanwhile, TE-SCALE incorporates an auto-suggestion feature, which returns relevant terms even based on partial inputs, further improving the search experience for users.

TE-SCALE promotes the widespread use of pan-cancer TE expression resources by ensuring seamless and convenient access for users. All data are openly accessible, allowing users to query and download relevant single-cell TE data on the “Download” page. This facilitates flexible data retrieval and diverse exploration to advance TE-related research.

### Interactive and functional exploration of tissue-level TE dynamics

For tissue-contextual exploration of TE expression, TE-SCALE integrates single-cell transcriptomic data across cancer types from shared tissue origins and provides an interactive UMAP explorer on the “Tissue Map” page. To enhance user experience, representative metacells were constructed from the original datasets, enabling efficient visualization while retaining essential biological signals. Researchers can interactively examine the expression patterns of specific genes or TE subfamilies to uncover potential co-expression relationships. The integrated atlas also includes rich cellular metadata, allowing flexible filtering and comparison by cell type, cancer type, donor characteristics (e.g. gender and age), and clinical information (e.g. tumor status, sampling site, and stage).

Notably, TE-SCALE offers functional analysis modules to support downstream investigation of TEs in tumor cells, including differential expression, TE-gene co-expression networks, and TE-related functional enrichment analysis. These tools help users interrogate TE dynamic features in the TME and gain valuable insights into their roles in cancer progression.

*Differential TE expression analysis*. TE-SCALE identifies TE subfamilies that are significantly upregulated in tumor cells compared with normal cells of the same tissue origin, using both single-cell and pseudobulk differential expression strategies. This module also performs differential TE expression analysis across tumor stages within a single cancer subtype for datasets with clinical stage information. Results are presented in an interactive table, where users can easily search, filter, and download the candidate lists. A heatmap is provided to visualize differential TE expression across cancer subtypes or tumor stages, with an overview of TE class composition.

*TE-gene co-expression network analysis*. TE-SCALE performs WGCNA on high-dimensional transcriptomic profiles of tumor cells to identify modules of highly co-expressed genes and TEs. Users can explore hierarchical clustering dendrograms of these co-expression modules alongside their expression patterns across different cell types, especially in tumor cells. For each module, TE-SCALE provides a detailed list and summary statistics of regulatory genes and TEs, including cancer-related genes annotated as oncogenes, TSGs, candidate cancer drivers, and tumor-specific TEs. Tumor-specific hub elements are identified, and gene-TE correlations are visualized as a network highlighting those with strong eigengene-based connectivity.

*Functional enrichment analysis*. Despite the poorly understood roles of most TE subfamilies in cancer, genes and TEs with similar expression patterns are often involved in related biological processes or regulatory networks. For further study, TE-SCALE performs functional enrichment analyses for each co-expression module: hallmark gene set enrichment helps infer putative pathways associated with TEs, whereas TE family enrichment reveals groups of TEs sharing structural and regulatory similarities. These analyses enable users to identify key TEs, explore their potential functions, and gene regulatory interactions in cancer development.

**Figure 4.**
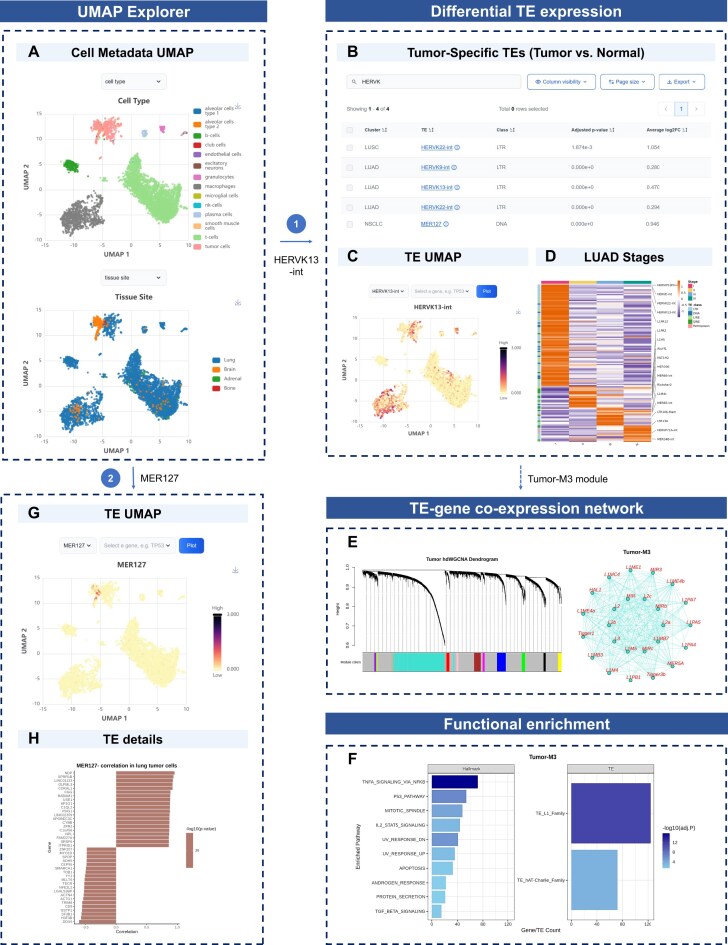
Case study of the “Tissue Map” exploration modules in TE-SCALE for lung cancer. (**A**) UMAP visualization of the integrated single-cell atlas of lung cancer, colored by cell type (top) and tumor site (bottom). (**B**) Tumor-specific TEs identified by differential TE expression analysis. (**C**) UMAP visualization of HERVK13-int expression. (**D**) Heatmap of stage-specific TE expression in LUAD. (**E**) TE-gene co-expression network analysis in tumor cells using WGCNA, showing the dendrogram of all modules (left) and the Tumor-M3 network (right). (**F**) Functional enrichment analysis of the Tumor-M3 module, including gene-based canonical pathways and TE family enrichment. (**G**) UMAP visualization of MER127 expression. (**H)** MER127 provides detailed information on correlated genes in lung tumor cells.

*Case study for lung cancer*. Transcriptional activation of human endogenous retroviruses (HERVs), particularly HERV-K, has been observed in a variety of tumors [18, 74, 75]. Using lung cancers as an example, TE-SCALE enables in-depth TE exploration through the UMAP explorer, displaying cell clustering by cell type, tumor site, and other related metadata (Fig. 4A). The “Differential TE expression” module identifies HERVK13-int, a HERV-K subfamily [76], as upregulated in tumor cells compared with normal epithelial cells, with elevated expression specifically in stage I lung adenocarcinoma (LUAD) tumors (Fig. 4B–D). In the “TE-gene co-expression network” module, WGCNA places HERVK13-int in Tumor-M3, a module enriched for 18.9% cancer-related genes, with the “Functional enrichment” highlighting pathways such as TNFα-NFκB signaling, p53, and apoptosis (Fig. 4E–F). These results indicate that HERVK13-int may serve as an early-stage diagnostic marker and contribute to potential tumor-immune interactions. Another example, MER127, is identified as a tumor-specific TE with selective upregulation in brain metastatic tumor cells (Fig. 4G). TE–gene correlation analysis links MER127 to SPRR1B, a LUAD-upregulated gene involved in proliferation, migration, and invasion [77] (Fig. 4H), suggesting its involvement in cancer metastasis. Together, these examples highlight TE-SCALE’s utility in uncovering tumor-specific TEs and their co-expression networks, which in turn facilitates mechanistic studies, biomarker discovery, and prioritization of tumor neoantigens.

### Pan-cancer analysis and clinical resource on tumor-specific TEs

**Figure 5.**
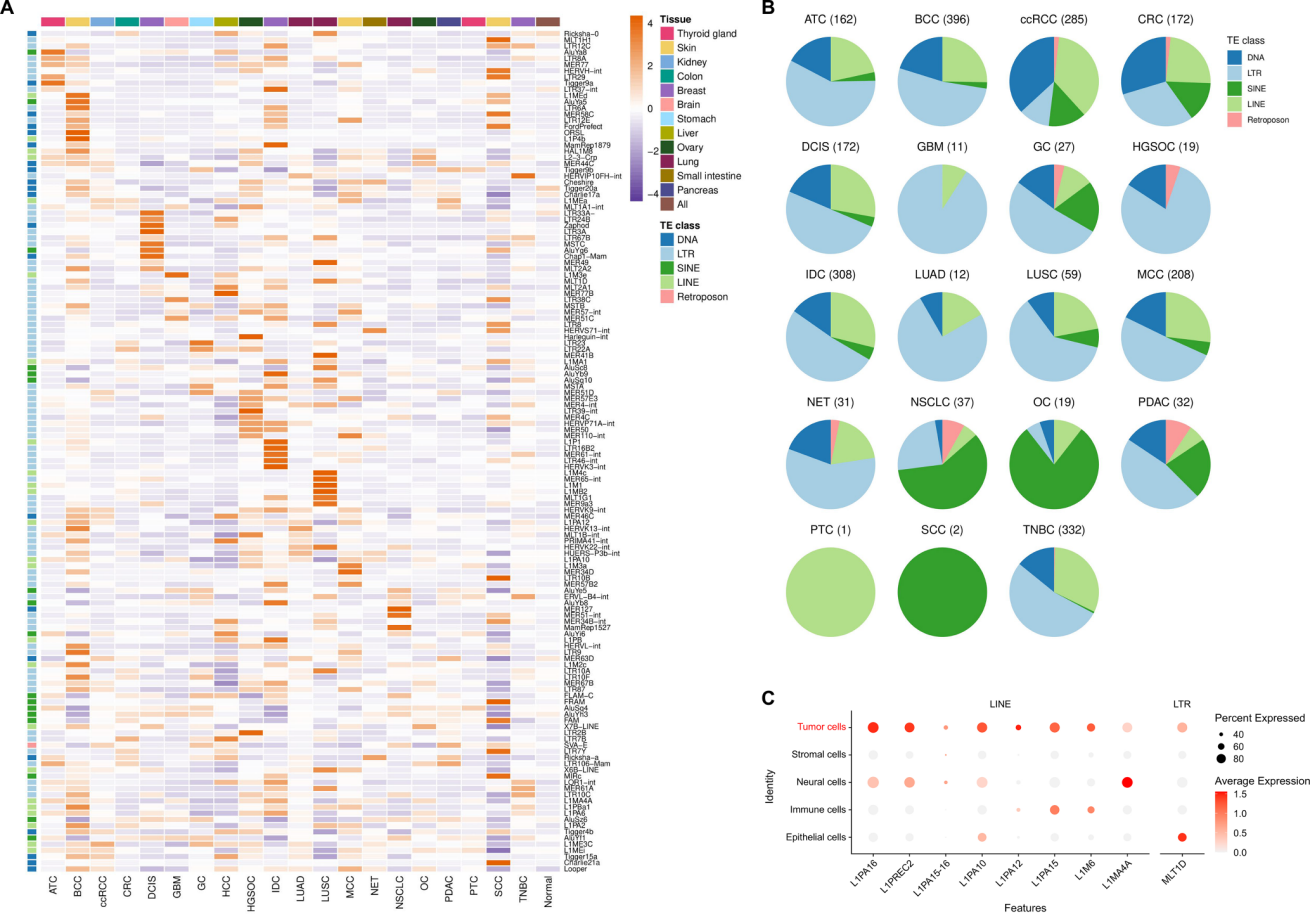
Pan-cancer analysis of tumor-specific TEs in TE-SCALE. (**A**) Expression of the top 20 tumor-specific TE subfamilies in tumor and normal cells across tissues, demonstrating clear cancer-type specificity. (**B**) TE class composition of tumor-specific subfamilies across cancer types. “Retroposon” here refers to SVAs. (**C**) Cancer-shared tumor-specific TE subfamilies expression, highlighting their potential as pan-cancer biomarkers and therapeutic targets. Cancer types: ATC, anaplastic thyroid carcinoma; BCC, basal cell carcinoma; ccRCC, clear cell renal cell carcinoma; CRC, colorectal cancer; DCIS, ductal carcinoma *in situ*; GBM, glioblastoma; GC, gastric cancer; HGSOC, high-grade serous ovarian cancer; IDC, invasive ductal carcinoma; LUAD, lung adenocarcinoma; LUSC, lung squamous cell carcinoma; MCC, Merkel cell carcinoma; NET, neuroendocrine tumor; NSCLC, non-small cell lung cancer; OC, ovarian cancer; PDAC, pancreatic ductal adenocarcinoma; PTC, papillary thyroid carcinoma; SCC, squamous cell carcinoma; TNBC, triple-negative breast cancer.

TE-SCALE systematically compiled tumor-specific TE identification results across 20 cancer types on the “Pan Cancer” page. Numerous TEs show clear cancer-type specificity (Fig. 5A). Remarkably, 148 TE subfamilies were uniquely specific to a single cancer type (Supplementary Figure S2). Among them, members of the HERVs and other LTRs serve as major tumor marker TEs across cancers (Fig. 5B). There is also a set of tumor marker TEs shared in different cancers. Most of these belong to the LINE-1 family and may serve as pan-cancer biomarkers and therapeutic targets (Fig. 5C).

Besides, TE-SCALE incorporates a curated collection of TE-related clinical studies, covering applications such as vaccines, antibody therapies, cell-based therapies, and neoantigen identification. Together, these computational findings and literature resources provide users with a valuable repertoire of candidate TEs to facilitate cancer diagnosis, immunotherapeutic target development, and disease progression monitoring.

### Discussion and future developments

Transposable elements harbor extensive regulatory potential that shapes the genomic and epigenomic evolution of cancer [18], exhibiting context-specific activation across diverse tumor types. Despite growing recognition of their biological significance and therapeutic relevance, a dedicated resource for systematically exploring pan-cancer TE expression dynamics and their regulatory implications at single-cell resolution has been lacking. To address this issue, we developed TE-SCALE, the first comprehensive database to integrate large-scale single-cell transcriptome data for profiling TE expression across human cancers. Through accurate TE quantification, in-depth single-cell analyses, and an interactive multidimensional visualization framework, TE-SCALE provides a user-friendly platform for researchers to investigate the roles of TEs in tumorigenesis from both cellular mechanistic and clinical perspectives.

Compared to existing tumor TE resources, TE-SCALE offers the broadest coverage of TE subfamilies, the highest resolution through single-cell data, and the most extensive dataset for cancer-related TE expression to date. Its distinguishing features include the following: (i) systematic and interactive exploration of TE expression across multiple levels, including pan-cancer, cancer/tissue, individual sample, and single cell; (ii) identification of tumor highly specific TE subfamilies, including both previously reported and novel elements; (iii) construction of TE-gene co-expression networks and modules in tumor cells, enabling inference of TE’s potential functions and (iv) a streamlined tool for single-cell TE subfamily quantification, scTEfinder.

As a featured database in the National Genomics Data Center (NGDC, https://ngdc.cncb.ac.cn), TE-SCALE will be regularly updated with newly released single-cell datasets. For instance, CancerSCEM, one of the key data sources for TE-SCALE, has recently been upgraded to version 2.0, encompassing 1466 scRNA-seq datasets across 74 cancer types [38]. The integration of it has been prioritized in the future update plan to further expand the scale of TE-SCALE. In summary, future expansions will include integration of additional cancer types, extension to non-tumor diseases, and enhanced annotation of clinical metadata, thereby improving data comprehensiveness and translational utility. Despite computational improvements in TE quantification, their accuracy and resolution remain constrained by the inherent limitations of short-read sequencing technologies, particularly considering the highly repetitive nature of TEs [47, 78]. Future development of integrative approaches leveraging long-read sequencing data holds promise for achieving precise genomic localization and quantification of TEs, enabling accurate dissection of *cis*/trans regulatory mechanisms, TE-expression quantitative trait locus (QTL) analysis, and determination of whether a TE target gene contains a cognate TE copy that could be co-expressed through co-transcription [79–81]. Such advancements will be incorporated into future updates of TE-SCALE. In addition, with the advancement of spatial transcriptomics technologies, it has become technically feasible to explore spatially specific expression patterns of TEs in diseases. For example, Braulio *et al.* developed SpatialTE, a pipeline that enables spatially resolved quantification of TE expression using 10x Genomics Visium data. They found that TEs exhibited spatially altered expression patterns in amyotrophic lateral sclerosis (ALS), as well as in mouse brain and kidney tissues [82]. Another study showed that TEs were enriched in tumor regions of gastric cancer (GC) tissue sections, indicating that enhanced TE expression might be a hallmark of GC. Furthermore, the authors found that TE activation in the TME might also play a role in GC initiation and progression [83]. Therefore, the investigation of spatially specific TE expression patterns across diseases will also be a future upgrade direction for TE-SCALE.

Leveraging TE-SCALE, researchers can systematically identify TE transcripts that are highly expressed or recurrent across tumors, yielding candidate tumor-specific TE-derived antigens (TS-TEAs) as well as potential biomarkers. These candidates have been preliminarily prioritized and functionally assessed in our database. They may be translated into clinical applications, such as molecular diagnostics for early detection or treatment monitoring [84, 85], and predictive models for prognosis [86, 87]. In future versions of TE-SCALE, survival analysis of TEs will be supported to explore their prognostic relevance. Although protein-level validation, including human leukocyte antigen (HLA) binding assays and mass spectrometry, remains to be performed, these TE-derived candidates offer promising avenues for tumor-specific diagnostics, prognostic indicators, and immunotherapy targets, highlighting TE-SCALE’s translational potential in precision oncology.

In conclusion, TE-SCALE provides a unique perspective for interrogating the “dark matter” of the genome by enabling comprehensive exploration of TE biology in cancer. It aims to establish a robust framework for translating TE-centric insights into clinical applications, including the development of novel diagnostic biomarkers and targeted therapeutic strategies in precision oncology.

## Supplementary Material

gkaf1235_Supplemental_File

## Data Availability

TE-SCALE is a comprehensive database for exploring TE expression across human cancers at single-cell resolution. The database is freely accessible at https://ngdc.cncb.ac.cn/te-scale. The scTEfinder is freely accessible at Zenodo (https://doi.org/10.5281/zenodo.17396260) or the GitHub website (https://github.com/synnimeng/scTEfinder).
