# Supplementary material for: TE-SCALE: a comprehensive database for exploring transposable element expression across human cancers at single-cell resolution
Source: Nucleic Acids Res. 2025 Nov 26;54(D1):D1658–71. doi: 10.1093/nar/gkaf1235 (PMC12807651; doi:10.1093/nar/gkaf1235)
Supplement: gkaf1235_Supplemental_File [file gkaf1235_supplemental_file.pdf]

Supplementary Table S1. Summary of scRNA-seq data collected across human cancers.

Tissue origin	Cancer type	Abbreviation	Sample #	Cell # (after QC)	Platform	Data source	Accession #	PMID	Publication
Brain	Glioblastoma	GBM	3	13024	10x 3' v2	CancerSCEM (GEO)	PRJNA579895	32004492	Adult Human Glioblastomas Harbor Radial Glia-like Cells
Breast	Normal human breast	Normal	16	53077	10x 3' v2	GEO	PRJNA749859	35617956	A human breast atlas integrating single-cell proteomics and transcriptomics
Colon	Colorectal cancer and adjacent normal tissue	CRC, normal	26	38631	10x 3' v2	CancerSCEM (ArrayExpress)	E-MTAB-8410	32451460	Lineage-dependent gene expression programs influence the immune landscape of colorectal cancer
Breast, thyroid gland	Triple-negative breast cancer, invasive ductal carcinoma, anaplastic thyroid cancer	TNBC, IDC, ATC	13	41981	10x 3' v2, 10x 5'	CancerSCEM (GEO)	PRJNA625321	33462507	Delineating copy number and clonal substructure in human tumors from single-cell transcriptomes
Breast, lung	Triple-negative breast cancer, non-small cell lung cancer	TNBC, NSCLC	4	29409	10x 3' v2	CancerSCEM (GEO)	PRJNA600483	39311908	Single-cell map of diverse immune phenotypes in the metastatic brain tumor microenvironment of non-small-cell lung cancer
Kidney	Clear cell renal cell carcinoma	ccRCC	4	46421	10x 3' v3	GEO	PRJNA768891	35853872	Single-cell multiomics analysis reveals regulatory programs in clear cell renal cell carcinoma
Kidney	Normal human kidney	Normal	3	14451	10x 3' v2	GEO	PRJNA544431	31896769	Single-cell RNA sequencing of human kidney
Liver	Hepatocellular carcinoma	HCC	2	10454	10x 3' v2	CancerSCEM (GEO)	PRJNA421273	30068984	High-density single cell mRNA sequencing to characterize circulating tumor cells in hepatocellular carcinoma
Lung	Lung adenocarcinoma, lung squamous cell carcinoma, non-small cell lung cancer and adjacent normal tissue	LUAD, LUSC, NSCLC, normal	18	45553	10x 3' v1/v2	CancerSCEM (ArrayExpress)	E-MTAB-6149	29988129	Phenotype molding of stromal cells in the lung tumor microenvironment
Lung	Lung adenocarcinoma, lung squamous cell carcinoma, non-small cell lung cancer and adjacent normal tissue	LUAD, LUSC, NSCLC, normal	12	25813	10x 3' v2	ArrayExpress	E-MTAB-6653	29988129	Phenotype molding of stromal cells in the lung tumor microenvironment
Lung	Lung adenocarcinoma	LUAD	17	43064	10x 3' v2	CancerSCEM (GEO)	PRJNA510251	32042191	Regenerative lineages and immune-mediated pruning in lung cancer metastasis
Lung	Lung adenocarcinoma	LUAD	55	410296	10x 3' v2	WCHSCU (GSA-Human)	PRJCA010830	38554705	Multicellular ecotypes shape progression of lung adenocarcinoma from ground-glass opacity toward advanced stages
Ovary	Ovarian cancer	OC	4	19178	10x 3' v3	CancerSCEM (ArrayExpress)	E-MTAB-8559	32054838	A living biobank of ovarian cancer ex vivo models reveals profound mitotic heterogeneity
Ovary	High-grade serous ovarian cancer and adjacent normal tissue	HGSOC, normal	8	31749	10x 3' v3	GEO	PRJNA754050	37100807	Overcoming adaptive resistance to anti-VEGF therapy by targeting CD5L
Pancreas	Pancreatic ductal adenocarcinoma and adjacent normal tissue	PDAC, normal	35	146648	10x 3' v2	CancerSCEM (GSA)	PRJCA001063	31273297	Single-cell RNA-seq highlights intra-tumoral heterogeneity and malignant progression in pancreatic ductal adenocarcinoma
Pancreas	Normal human pancreatic islet	Normal	14	79418	10x 3' v2	GEO	PRJNA761336	34428183	Combinatorial transcription factor profiles predict mature and functional human islet α and β cells
Skin	Basal cell carcinoma, squamous cell carcinoma	BCC, SCC	48	96699	10x 5'	CancerSCEM (GEO)	PRJNA509910	31359002	Clonal replacement of tumor-specific T cells following PD-1 blockade
Skin	Merkel cell carcinoma	MCC	2	8170	10x 3' v2	CancerSCEM (GEO)	PRJNA483959	30250229	Acquired cancer resistance to combination immunotherapy from transcriptional loss of class I HLA
Skin	Merkel cell carcinoma	MCC	2	13083	10x 5'	CancerSCEM (GEO)	PRJNA484204	30250229	Acquired cancer resistance to combination immunotherapy from transcriptional loss of class I HLA
Skin	Normal human skin	Normal	5	21459	10x 3' v2	GEO	PRJNA542149	32327715	Single-cell transcriptomes of the human skin reveal age-related loss of fibroblast priming
Small instestine	Neuroendocrine Tumor	NET	2	4556	10x 3' v2	CancerSCEM (GEO)	PRJNA589287	32054662	Comparative single-cell RNA sequencing (scRNA-seq) reveals liver metastasis-specific targets in a patient with small intestinal neuroendocrine cancer
Stomach	Gastric cancer and adjacent normal tissue	GC, normal	4	6327	10x 3' v2	CancerSCEM (GEO)	PRJNA555477	31067475	Dissecting the Single-Cell Transcriptome Network Underlying Gastric Premalignant Lesions and Early Gastric Cancer
Thyroid gland	Anaplastic thyroid cancer, papillary thyroid carcinoma and adjacent normal tissue	ATC, PTC, normal	23	68798	10x 3' v3	GEO	PRJNA796654	37053016	Anaplastic transformation in thyroid cancer revealed by single-cell transcriptomics
Thyroid gland	Anaplastic thyroid cancer, papillary thyroid carcinoma and adjacent normal tissue	ATC, PTC, normal	10	49544	10x 3' v2	GSA-Human	PRJCA003390	34321197	Characterizing dedifferentiation of thyroid cancer by integrated analysis

Supplementary Table S2. Summary of studies using TEs as immunotherapy targets.

Application	Target TE or gene	Cancer type	Abbreviation	Model system	Immunogenicity	PMID	Publication
Vaccine	gp70 or p15E from ERVs	Colorectal carcinoma, melanoma	CRC, MM	Mouse in vivo	Yes	11691813	Immunization against Endogenous Retroviral Tumor-associated Antigens
	HERV-E	Clear cell renal cell carcinoma	ccRCC	Human in vitro	Yes	30137025	Endogenous retroviral signatures predict immunotherapy response in clear cell renal cell carcinoma
	JET-derived peptides	Melanoma, colorectal carcinoma, sarcoma	MM, CRCC, SARC	Mouse in vivo	Yes	36735776	Epigenetically controlled tumor antigens derived from splice junctions between exons and transposable elements
Antibody	ERVK-7 or HERV-K102	Lung adenocarcinoma	LUAD	Human and mouse in vivo	Yes	37046094	Antibodies against endogenous retroviruses promote lung cancer immunotherapy
	TS-TEPs such as L1PA2_GABRG2	Pan-cancer	Pan	Human in vitro	Not reported	36973455	Pan-cancer analysis identifies tumor-specific antigens derived from transposable elements
CAR-T cells	HERV-K env	Melanoma	MM	Human cells and mouse xenograft	Yes	25829402	Genetic Engineering of T Cells to Target HERV-K, an Ancient Retrovirus on Melanoma
	RN7SL1	Pancreatic cancer, melanoma	PDAC, MM	Mouse in vivo	Yes	34464586	The immunostimulatory RNA RN7SL1 enables CAR-T cells to enhance autonomous and endogenous immune function
Neoantigen	CT-RCC-1 derived from HERV-E	Renal cell carcinoma	RCC	Human in vitro	Yes	18292810	Regression of human kidney cancer following allogeneic stem cell transplantation is associated with recognition of an HERV-E antigen by T cells
	HERV-K env	Breast cancer	BC	Human in vitro	Yes	18632641	Human Endogenous Retrovirus K Triggers an Antigen-Specific Immune Response in Breast Cancer Patients
	HERV-H ORF on Xp22.3	Colorectal carcinoma	CRC	Human in vitro	Yes	22187063	Endogenous retrovirus sequences as a novel class of tumor-specific antigens: an example of HERV-H env encoding strong CTL epitopes
	HERV-E env	Clear cell renal cell carcinoma	ccRCC	Human in vitro	Yes	26862115	Detection of an Immunogenic HERV-E Envelope with Selective Expression in Clear Cell Kidney Cancer
	ERV9, LTR12	Lung cancers	LC	Human cells	Yes	28604729	DNMT and HDAC inhibitors induce cryptic transcription start sites encoded in long terminal repeats
	HERV 4700	Clear cell renal cell carcinoma	ccRCC	Human in vitro	Yes	30137025	Endogenous retroviral signatures predict immunotherapy response in clear cell renal cell carcinoma
	SVA_D and LTR12C	Pan-cancer, glioblastoma	Pan, GBM	Human in vitro	Not reported	31745090	Transposable element expression in tumors is associated with immune infiltration and increased antigenicity
	THE1A, MLT1B, MLT1A0	Pan-cancer	Pan	Human in vitro	Not reported	31537638	LTR retroelement expansion of the human cancer transcriptome and immunopeptidome revealed by de novo transcript assembly
	AluJb-LIN28B	Pan-cancer, lung cancer	Pan	Human in vitro	Not reported	30926969	Transposable elements drive widespread expression of oncogenes in human cancers
	Inverted-repeat Alu elements	Pan-cancer	Pan	Human cells and mouse xenograft	Yes	33087935	Epigenetic therapy induces transcription of inverted SINEs and ADAR1 dependency
	HERVH-5, HERVW-1, HERVE-3	Hematological cancers	HC	Human in vitro	Yes	33168830	Human endogenous retroviruses form a reservoir of T cell targets in hematological cancers
	A subset of HERV elements	Pan-cancer, triple-negative breast cancer	Pan, TNBC	Human in vitro organoid	Yes	35080970	Identification of shared tumor epitopes from endogenous retroviruses inducing high-avidity cytotoxic T cells for cancer immunotherapy
	Non-redundant peptide-coding TEs	Glioblastoma	GBM	Human in vitro	Not reported	35675780	Single-cell RNA-seq-based proteogenomics identifies glioblastoma-specific transposable elements encoding HLA-I-presented peptides
	A subset of HERV elements	Acute myeloid leukemia	AML	Human in vitro	Yes	35759575	HERVs characterize normal and leukemia stem cells and represent a source of shared epitopes for cancer immunotherapy
	JET-derived peptides	Non-small cell lung cancer	NSCLC	Human in vitro	Yes	36735774	Noncanonical splicing junctions between exons and transposable elements represent a source of immunogenic recurrent neo-antigens in patients with lung cancer
	TS-TEAs	Pan-cancer	Pan	Human in vitro	Not reported	36973455	Pan-cancer analysis identifies tumor-specific antigens derived from transposable elements
	LTR12-derived TINPATs	Acute myeloid leukemia	AML	Human cells and in vitro	Yes	37872136	DNMT and HDAC inhibition induces immunogenic neoantigens from human endogenous retroviral element-derived transcripts
	HERV 3895	Renal cell carcinoma	RCC	Human in vitro	Yes	37606040	Proteogenomic identification of an immunogenic antigen derived from human endogenous retrovirus in renal cell carcinoma
	LTR12, THE1	Glioblastoma	GBM	Human cells and in vitro	Yes	39223316	Epigenetic therapy potentiates transposable element transcription to create tumor-enriched antigens in glioblastoma cells

Note: TEs or genes listed in the ‘Neoantigen’ rows were not applied in therapy, and their potential remains to be explored. TINPATs, treatment-induced novel polyadenylated transcripts; TS-TEAs, tumour-specific TE-chimeric antigens; TS-TEPs, tumour-specific TE-chimeric proteins.

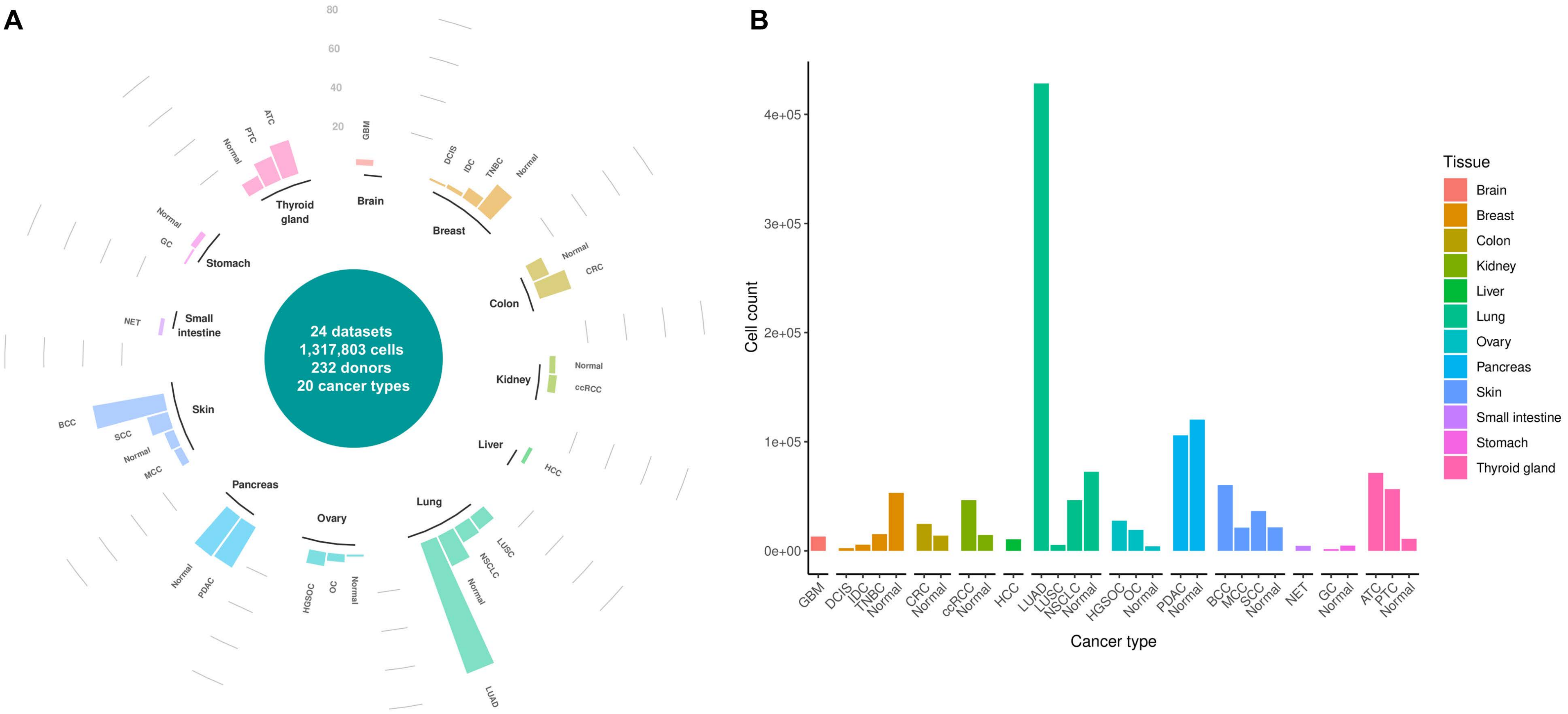

Supplementary Figure S1. Statistics of the sample (A) and cell (B) distribution across cancer types.

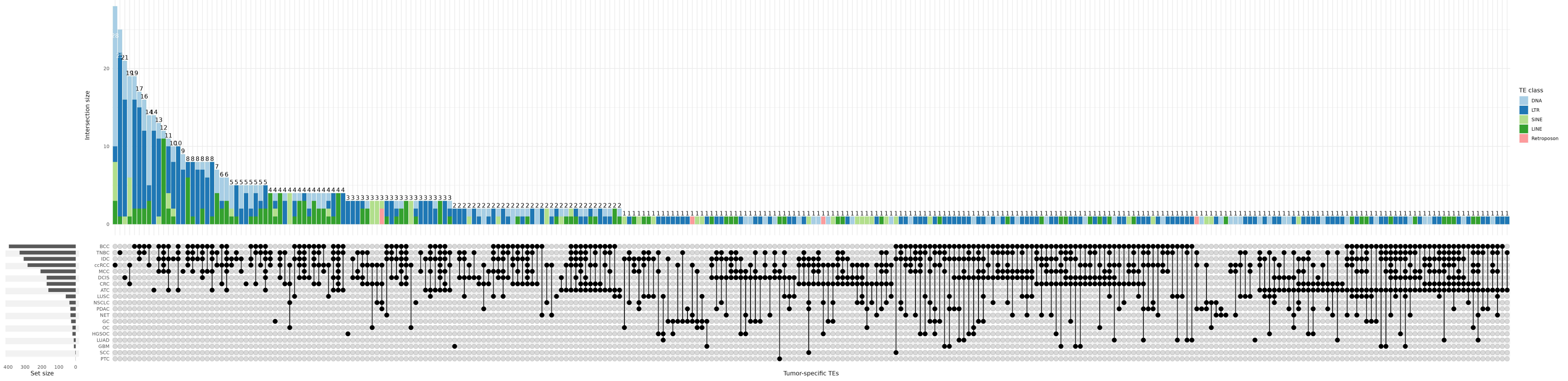

Supplementary Figure S2. Upset plot showing the sharing and specificity of all tumor-specific TE subfamilies across cancer types.
